# Functional Foods and Dietary Bioactives in Human Health

**DOI:** 10.3390/molecules31142496

**Published:** 2026-07-17

**Authors:** Raffaella Comitato, Federica Intorre, Eugenia Venneria

**Affiliations:** 1 Consiglio per la ricerca in agricoltura e l’analisi dell’economia agraria—Council for Agricultural Research and Economics (CREA), Research Centre for Cereal and Industrial Crops, via Torrino 2, 81100 Caserta, Italy; 2 Consiglio per la ricerca in agricoltura e l’analisi dell’economia agraria—Council for Agricultural Research and Economics (CREA), Research Centre for Food and Nutrition, Via di Vigna Murata 605, 00143 Rome, Italy; federica.intorre@crea.gov.it (F.I.); eugenia.venneria@crea.gov.it (E.V.)

The study of functional foods and dietary bioactives has matured to a point where the central question is no longer whether food components influence human physiology, but how they do so and, increasingly, through how many routes at once. The rising global burden of non-communicable diseases has kept dietary strategies at the centre of preventive research, yet the most informative recent work shares a feature that is easy to overlook: the molecules that matter rarely act alone or on a single target. This Special Issue of *Molecules*, entitled “Functional Foods and Dietary Bioactives in Human Health”, gathers contributions that together trace a shift in how the field frames mechanisms. They invite us to treat bioactives less as isolated agents with a defined effect and more as elements of interacting systems, combinations of compounds engaging combinations of targets.

This framing is worth stating explicitly because it reorganizes how the individual papers relate to one another. The seven contributions collected here span chemical characterization of plant matrices, computational and cellular interrogation of molecular targets, metabolic synergy in muscle cells, dietary modulation of lipoprotein metabolism, and the valorization of agri-food by-products into functional products. Their diversity is real, but what unites them is an implicit move away from the one-molecule, one-effect logic that long organized nutritional biochemistry, toward a logic of combination, network, and context.

**From compound to combination**

At the biological level, the bioactives examined across this collection act through antioxidant, anti-inflammatory, and immunomodulatory routes and through the regulation of metabolic and signalling pathways tied to chronic disease. These are not separate effects to be listed but converging modes of action, and combining them together is what reveals the pattern. Two of the studies make the point explicit by placing synergy at the centre rather than at the margin.

The work on oxaloacetate and ketone bodies in L6 myoblasts shows that two metabolites, neither remarkable on their own at the concentrations used, together promote myoblast differentiation more effectively than either alone [Contribution 1]. This is not an incidental observation but the key finding: it reframes dietary metabolites as partners whose joint action defines the physiological outcome, a perspective with direct relevance to muscle maintenance and to the metabolic dimension of ageing.

The comparative molecular dynamics study of food-derived flavonoids as PD-L1 inhibitors extends the same logic in a different direction [Contribution 2]. By screening sixty flavonoids across six structural subgroups against the PD-L1 dimer through docking and dynamics simulations and then confirming that the leading candidates inhibit cancer cell proliferation in a cell-based assay, the authors do more than nominate ginkgetin and diosmin as strong binders: they map how subtle differences in scaffold (flavonol versus flavone, isoflavone versus anthocyanidin) translate into measurable differences in binding behaviour at a single immuno-oncological target. The compound is no longer a point estimate of activity: it is a position within a structural space, and that space is what determines function. This is structure–activity reasoning carried to the level where a class of natural products meets a defined therapeutic target.

**From matrix to repertoire**

If the first pair of papers discusses compounds as combinations, a second group understands the food matrix itself as a repertoire of bioactives rather than a passive carrier. The comprehensive overview of plant-derived terpenoids assembles a striking range of structures and functions, presenting this class not as a catalogue but as a functional reservoir whose breadth is precisely what makes it valuable across health and industrial applications [Contribution 3]. The characterization of sea buckthorn leaves goes beyond the level of a single matrix, documenting a composition rich and varied enough that the leaf is best understood as a system of co-occurring actives rather than a source of one headline compound [Contribution 4].

This understanding also draws attention to the physicochemical and functional properties of bioactive compounds within complex food systems. Structural characteristics such as molecular size, conformation, and interaction with other matrix components shape not only technological behaviour (stability, rheology, texture) but also biological activity and bio-efficacy themselves. Functional foods are, in this sense, complex systems in which the interactions between components can significantly affect the final health outcome, and food chemistry and molecular nutrition have to be considered together to understand how they behave in real dietary contexts.

The study of bioaccessibility of phenolic compounds, resistant starch, and dietary fibres from Australian green banana during in vitro digestion and colonic fermentation sharpens this point by following the matrix into the gut [Contribution 5]. The effectiveness of a dietary bioactive depends not only on its intrinsic activity but on its release from the matrix during digestion, its transformation, and its interaction with the host. What reaches the colon, and in what form, is therefore an emergent feature of the matrix in transit rather than a property of any molecule in isolation. Advances in in vitro digestion models, applied here to real food, are precisely what allow this dynamic repertoire to be observed and the gap between intake and physiological response to be narrowed.

**From mechanism to physiological outcome**

Two further contributions connect this network thinking to integrated physiological endpoints. The review of the effect of diet on HDL in obesity addresses lipoprotein metabolism not through a single nutrient but through dietary patterns acting on a system whose regulation is intrinsically multifactorial [Contribution 6]. HDL functionality in obesity is shaped by many converging inputs, including adipokines, oxidative stress, dietary patterns, and individual polyphenols, and the contribution is valuable for holding these together rather than isolating one. The valorization of red beetroot pomace combined with golden linseed into gluten-free vegetable crispbreads closes the arc on the applied side, turning agri-food by-products into a product whose nutritional interest rests on a combination of bioactive compounds delivered together in a food people might actually eat [Contribution 7]. This last study also speaks to a community for whom matrix composition is not academic: for those who must avoid gluten, the design of nutrient-dense alternatives from undervalued raw materials is a concrete contribution to dietary quality.

Across these seven studies, the methodological range itself is informative. Molecular docking and dynamics, targeted gene-expression analysis and cellular differentiation assays, simulated digestion and colonic fermentation, compositional characterization, and the synthesis of dietary evidence each illuminate a different layer of the same problem. Computational and analytical tools of this kind are what allow complex mixtures of bioactives to be characterized, and their candidate mechanisms resolved, helping the field move from descriptive toward more mechanistic and predictive research. No single method is sufficient on its own. The plurality of approaches—in silico, cellular, in vitro digestion, and dietary—mirrors the plurality of targets the bioactives engage, even though these contributions address independent questions rather than steps in a single mechanistic pipeline.

Several challenges remain, and the contributions are candid about them. Translating in vitro and preclinical findings into robust clinical evidence is still the critical step, and variability arising from food matrices, processing conditions, and individual responses limits the generalization of results. Addressing this will require standardized methodologies and genuine interdisciplinary collaboration across chemistry, biology, nutrition, and medicine—a need that is, in fact, one of the central messages of this collection.

**A network of targets implies a variability of response**

There is a consequence to taking the network view seriously, and it points to what we see as the next frontier for this field. If a bioactive acts not on one target but on several, and if its effect emerges from interactions within a biological system rather than from a fixed pharmacological property, then the response cannot be a constant: it must depend on the system responding. Two individuals carry different genetic and epigenetic configurations of the very pathways these compounds engage; the same flavonoid, the same terpenoid, and the same metabolite pair will therefore not produce the same outcome in both. Variability of response cannot be averaged away: it is the predictable signature of multi-target action.

This reframes the standardization challenge that rightly preoccupies the field. Much attention goes, correctly, to standardizing the product, securing batch-to-batch equivalence, reproducible composition, and controlled exposure. But a network of targets implies that we will eventually also need to account for the standardization of the response, which means understanding why the same intervention helps one person and not another. Nutrigenomic and epigenetic approaches, which investigate how dietary signals are received and interpreted at the level of gene regulation, are the natural instruments for this task. The studies in this Special Issue do not themselves stratify responders, and we do not claim they do; their contribution is to make the underlying logic visible. Once bioactives are understood as modulators of interacting pathways, individual variability follows as a matter of course, and the question of who responds, and why, moves from the margins of the discussion to its centre.

Taken together, the contributions assembled here offer a coherent and forward-looking picture. They move past the inventory of single molecules with single effects toward an understanding of functional foods as repertoires of compounds acting in combination on networks of targets, within matrices that shape exposure and organisms that shape response. We thank the authors for their work, the reviewers for their careful and constructive evaluation, and the editorial team for their support. We hope this collection encourages research that retains complexity rather than reducing it away, and that takes the variability of human response not as an inconvenience but as the most interesting thing the field has yet to explain.

